# Optimal Electrical Properties of Outer Hair Cells Ensure Cochlear Amplification

**DOI:** 10.1371/journal.pone.0050572

**Published:** 2012-11-27

**Authors:** Jong-Hoon Nam, Robert Fettiplace

**Affiliations:** 1 Department of Mechanical Engineering, Department of Biomedical Engineering, University of Rochester, Rochester, New York, United States of America; 2 Center for Navigation and Communication Sciences, University of Rochester Medical Center, Rochester, New York, United States of America; 3 Department of Neuroscience, University of Wisconsin-Madison, Madison, Wisconsin, United States of America; Stanford University School of Medicine, United States of America

## Abstract

The organ of Corti (OC) is the auditory epithelium of the mammalian cochlea comprising sensory hair cells and supporting cells riding on the basilar membrane. The outer hair cells (OHCs) are cellular actuators that amplify small sound-induced vibrations for transmission to the inner hair cells. We developed a finite element model of the OC that incorporates the complex OC geometry and force generation by OHCs originating from active hair bundle motion due to gating of the transducer channels and somatic contractility due to the membrane protein prestin. The model also incorporates realistic OHC electrical properties. It explains the complex vibration modes of the OC and reproduces recent measurements of the phase difference between the top and the bottom surface vibrations of the OC. Simulations of an individual OHC show that the OHC somatic motility lags the hair bundle displacement by ∼90 degrees. Prestin-driven contractions of the OHCs cause the top and bottom surfaces of the OC to move in opposite directions. Combined with the OC mechanics, this results in ∼90 degrees phase difference between the OC top and bottom surface vibration. An appropriate electrical time constant for the OHC membrane is necessary to achieve the phase relationship between OC vibrations and OHC actuations. When the OHC electrical frequency characteristics are too high or too low, the OHCs do not exert force with the correct phase to the OC mechanics so that they cannot amplify. We conclude that the components of OHC forward and reverse transduction are crucial for setting the phase relations needed for amplification.

## Introduction

The organ of Corti (OC) is the sensory epithelium unique to the mammalian cochlea. It is sandwiched between two tissues called the basilar membrane and the tectorial membrane, and these layers comprise the cochlear partition separating the two fluid compartments known as the scala media and scala tympani. The OC is composed of sensory receptor cells called hair cells and other supporting cells. Outer hair cells (OHCs) are thought to amplify vibrations to weak sounds to facilitate detection by the inner hair cells [1]. Unlike the structurally simpler auditory epithelia of lower vertebrates, the OC has a complex geometry that might be important for its kinematic gain [2] and the OC mechanics for cochlear amplification. Recent experimental observations have provided more details about of OC mechanics. For example, current applied across the OC resulted in opposite displacements of the top and bottom surfaces of the OC that was ascribed to voltage-dependent OHC motility [3]. High resolution confocal microscopy combined with image analysis captures the relative motion between the tectorial membrane and the reticular lamina [4]. Stroboscopic illumination and imaging demonstrated that the OC mechanics is highly complicated and dependent on the type of stimulation–acoustical or electrical [5], [6]. Optical coherence tomography provided a clearer view of the relative motion within the OC [7], [8] and showed that the vibration at the top surface of the OC leads the bottom by about 90 degrees at low stimulation levels, the phase difference diminishing as the stimulation level increases.

We have created an electro-mechanical model of the cochlear partition that explains the recent experimental observations including the difference in phase and peak frequency between the OC structures. The work further developed our fully deformable finite element structural model that embodies minimal kinematic assumptions [9]. While our previous work was largely a static analysis focused on deformation within the OC, the present work analyzes the phase relations (dynamics) within the OC by including the hair bundle mechano-transduction kinetics and the OHC somatic motility. For the electrical representation of the OHC, recently measured membrane properties were incorporated [10]. According to that work, the membrane filtering frequency of OHCs tuned to higher frequencies is more than one order of magnitude higher than was previously thought. With this new model, we explored the dynamic relations between the OC mechanical variables such as the relative displacements of the tectorial membrane and reticular lamina determining hair bundle motion, and the electrical variables such as the OHC receptor potential and contractility dictating the feedback. Our results suggest that forward and reverse transduction in the OHCs are necessary to set the phase of the feedback to achieve amplification.

## Results

**Figure 1:**
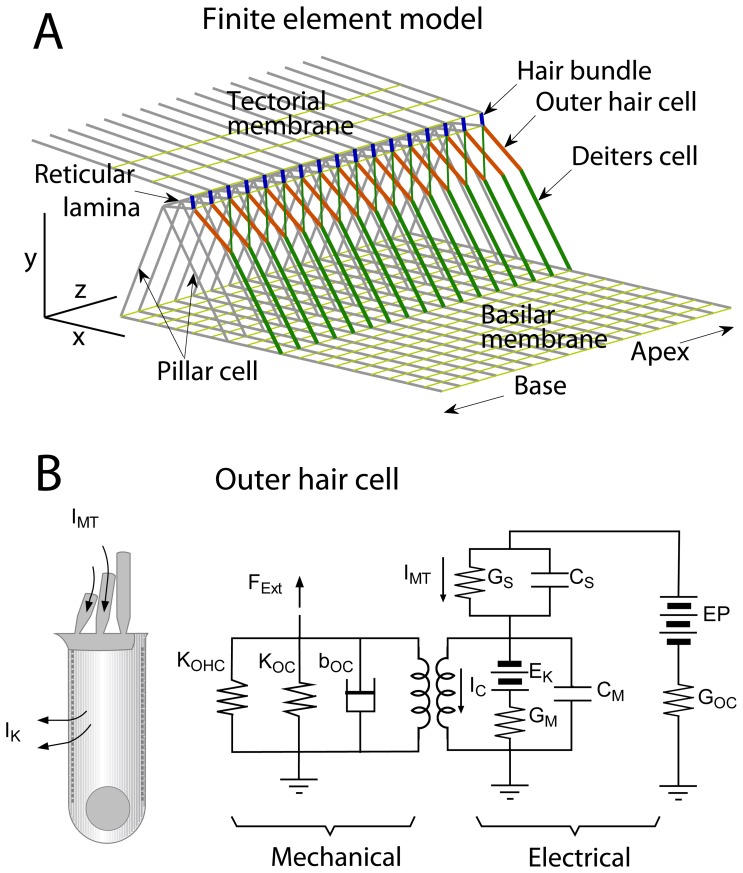
Cochlear partition model. (**A**) 3D finite element model of the cochlear partition. (**B**) Electrical representation of an OHC and its piezoelectricity. For illustrative purpose, the mechanical system around the OHC was expressed with *K_OHC_*, *K_OC_* and *b_OC_* that correspond to OHC stiffness, stiffness of OC without OHCs, and viscous damping in the OC. In the analysis, the finite elements in **A** represent the mechanical part. Key electrical parameters are maximum mechanotransducer conductance, *G_S_* = 27, 90 nS, K^+^ conductance across basolateral membrane, *G_M,max_* = 45, 370 nS at the two cochlear locations; Membrane capacitance of basolateral membrane, *C_M_*_,_ is six time greater than *C_S_*_,_ membrane capacitance of stereociliary membrane, *C_M_* = 15, 4.3 pF, for apex and base respectively; K^+^ equilibrium potential, *E_K_* = 70 mV and endocochlear potential, *EP* = 90 mV.

The elongated cochlea is tonotopically organized such that high sound frequencies are detected towards the basal end and low sound frequencies towards the apical end. Hereafter, the “apex” and the “base” denote the locations in the gerbil cochlea with best frequencies (BF) of near 0.6 kHz and 19 kHz respectively. The gerbil’s basilar membrane is 12 mm long and its audible frequency range extends from about 0.25 to 45 kHz [11]. The basal location, although having a BF at least an octave below the upper frequency limit for the gerbil, was chosen because it is the most basal location for which electrical and mechanical data exist. The x-axis and y-axis in the model correspond to the radial (neural-abneural) and transverse (normal to the basilar membrane plane) directions respectively (Fig. 1).

### Amplification by OHC–Impulse Response of the Cochlear Partition

**Figure 2:**
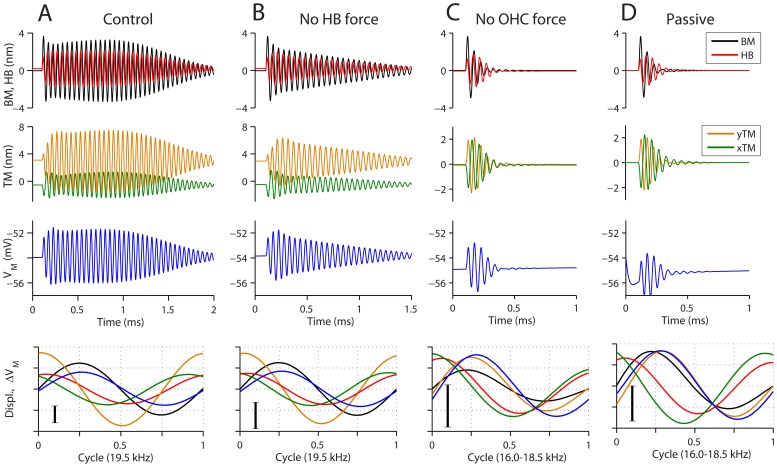
Impulse response of the cochlear partition. An impulse of 0.5 nN-ms was applied to the middle of the BM at t = 0.1 ms. Four cases are: (**A**) Control with both hair-bundle mechanotransduction force and somatic force; (**B**) hair bundle mechano-transduction force set to zero; (**C**) OHC somatic force set to zero; and (**D**) without active mechanical feedback from the OHCs. Top row: BM displacement, hair bundle shear displacement. Second row: TM displacements in the x- and y-direction (radial and transverse direction). Third row: OHC membrane voltage. Bottom row: the responses for one cycle were plotted together after subtracting the DC component. The vertical scale bars indicate 1 nm and 1 mV.

Amplification was primarily achieved by OHC somatic motility. In order to compare how two types of OHC active force, hair bundle force originating from mechano-transduction apparatus [12] and somatic force attributable to the membrane protein prestin [13], affect the cochlear partition vibration, four different cases were simulated (Fig. 2). The four cases are: control (with both somatic and hair bundle motors), without hair bundle force (only somatic force), without somatic force (only hair bundle force) and passive (without either force). The mechanotransduction channel kinetics and the transduction current were common to all four cases. A finite element model of the cochlear partition from the basal turn of gerbil cochlear coil, a 600 µm piece centered at ∼19 kHz BF, was simulated. The length of coil simulated is large compared to the space constants for longitudinal coupling measured in the gerbil cochlea [14]. When a 0.5 nN-ms impulse stimulus was applied to the basilar membrane, the cochlear partition vibrated at its BF, governed by the stiffness of the basilar membrane and the mass carried. In the control, it took 41 oscillating cycles in 2.1 ms before the oscillation dissipated below 5 percent of the peak. Without the hair bundle force, it took 32 cycles in 1.6 ms. When the OHC somatic force was not fed back to the OC mechanics, the oscillations dissipated within 4–6 cycles. Thus the somatic motor made by far the largest contribution to tuning and the hair bundle force amplification only when present together with the OHC somatic force. Similar results were obtained at the apex (data not shown) where, following an impulse stimulus, the apical section oscillated at 0.6 kHz. It took 9, 8, 5 and 5 oscillations before settling down below 5 percent of the peak for control, without hair bundle force, without OHC somatic force and without any force feedback from OHCs. Therefore, the amplification is dominated by OHC somatic motility at both apex and base.

In the basal section, about 40 percent of the OHC transducer channels were open at rest [10], which created a 5.1 nA resting or ‘silent’ transducer current. The balance between the resting transducer current and outwardly rectifying membrane potassium current resulted in a −54 mV resting membrane potential. After 1 ms of impulse application, one nanometer of basilar membrane displacement resulted in 0.65 mV OHC receptor potential (Fig. 2 bottom row). A single OHC in the middle of the simulated partition generated 6 pN force out of the mechano-transduction apparatus, and 60 pN out of the somatic motility per one nm basilar membrane displacement. The hair bundle force led the basilar membrane displacement by 61 degrees while the somatic force lagged by 7 degrees.

The inclusion of somatic force of the OHCs resulted in higher oscillation frequencies (19.5 kHz, Fig. 2A, B) than the other cases (16–19 kHz, Fig. 2C, D). All parts of the cochlear partition at a radial section (in the x-y plane) vibrated with the same frequency when the OHC somatic force was dominant (or when small stimulation was applied at the BF of the site). However, when no OHC somatic force was fed back to the OC (Fig. 2C and D), two different frequencies were excited immediately after the impulse and settled down to the lower frequency. In the two to three oscillations after the impulse, the tectorial membrane vibrated faster in the transverse direction (yTM oscillates at ∼20 kHz) than in the radial direction (xTM oscillates at ∼17 kHz).

**Figure 3:**
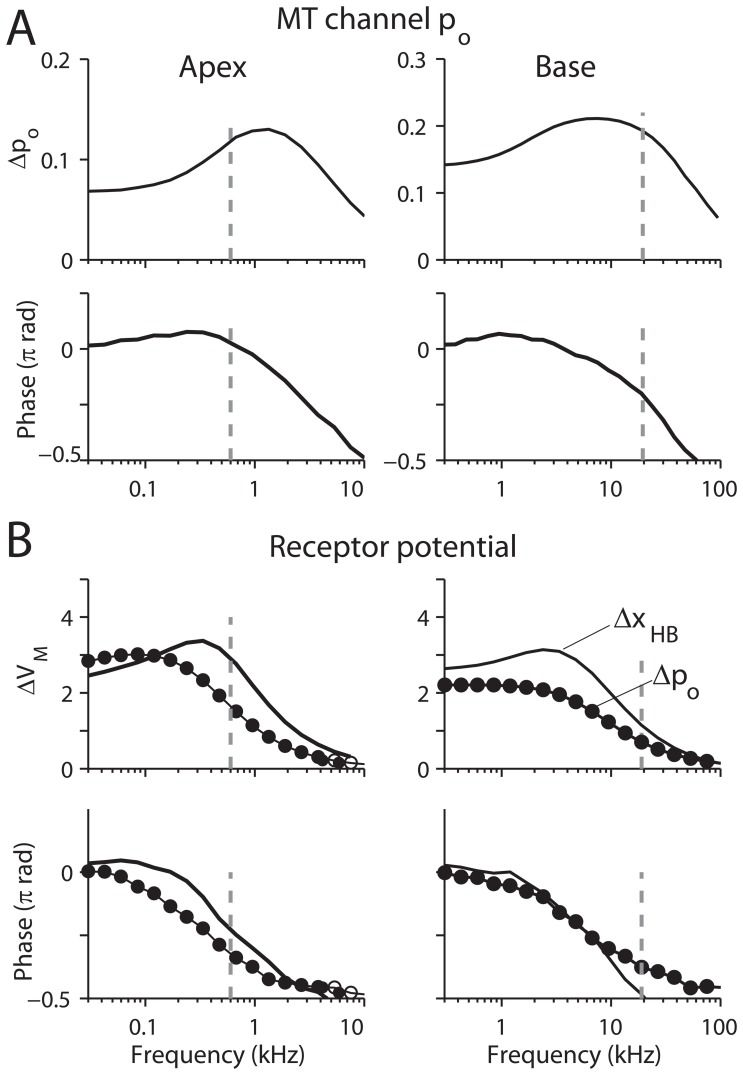
Response of a single OHC to hair bundle stimuli. (**A**) An OHC hair bundle was stimulated with a sinusoidal force applied at the tip of the bundle with amplitude 20 pN (left, apex) and 100 pN (right, base). Change in probability of opening of the transduction channel Δp_o_ (top) and phase with respect to the applied force (bottom) are plotted versus stimulation frequency. (**B**) An OHC was stimulated either by sinusoidal modulation of the mechano-transduction channel open probability about a mean value of 0.4 (•, *p_o_* = 0.4+0.05sin(*ωt*) ) or by sinusoidal hair bundle displacement (solid lines, *x_HB_* = *x_O_* sin(*ωt*) where *x_O_* = 3.3. nm, left apex, or *x_O_* = 1.0 nm, right, base) at different frequencies. The amplitude of the OHC receptor potential (*ΔV_M_*, (top) and its phase with respect to the stimulus (bottom) are shown. The BF at each OHC’s location (0.60 and 19 kHz) is indicated with broken vertical lines.

There was a notable phase difference between responses with and without OHC somatic motility (bottom row of Fig. 2). The transverse displacement of the tectorial membrane (yTM, orange line) led the basilar membrane displacement (yBM, black line) by ∼90 degrees only when the OHC somatic force was incorporated. Note that the phase of the tectorial membrane radial displacement (xTM, green line) was little affected by OHC motility. In order to explain how these phase relations are determined, we investigated the response of an individual OHC (Fig. 3).

### Response of an Isolated OHC

An individual OHC was simulated in order to observe the relationship between hair bundle displacement, transducer current and receptor potential (Fig. 3). To impose the proper mechanical impedance, we obtained the OC stiffness felt by the OHC from the finite element model, which was comparable to the OHC stiffness itself. Recently measured membrane electrical properties were used throughout this study (Table 1, [10]).

**Table 1:** Outer hair cell electrical properties.

Symbol	Apex	Base	Description	Reference
*EP* (mV)	90	90	Endocochlear potential	[41]
*E_k_* (mV)	75	75	Cell equilibrium potential	[42]
*G_S,max_* (nS)	27	90	Max. hair bundle conductance	[10]
*C_S_* (pF)	1/6 of *C_M_*		Hair bundle capacitance	[10]
*G_ M,max_* (nS)*	45	370	Max. cell membraneconductance	[10]
*C_M_* (pF)	11	6.4	Cell membrane capacitance	[10]

* After considering the voltage-dependence of the membrane conductance, G_M_ becomes 38 and 265 nS at the apex and the base respectively.

Three cases were tested. Firstly, the OHC hair bundle was stimulated with sinusoidal force at different frequencies and the amplitude of normalized transduction current was observed (Fig. 3A and B). The mechano-transduction apparatus produces a broad band-pass filter peaking at about 1 kHz and 10 kHz (apex and base respectively, Fig. 3A and B). Three factors shape this filter: activation and adaptation kinetics of the mechanotransducer channels and viscous damping on the hair bundle (Table S1 in Supporting Information S1). Secondly, a single OHC was electrically stimulated by modulating the mechanotransducer current to measure the membrane potential (Fig. 3C and D, lines with •).

A 5 percent modulation of the resting transduction current resulted in 3 mV and 2.5 mV OHC receptor potentials at the apex and base respectively. Because the membrane behaves as a first-order low pass filter, the phase lag develops from zero to 90 degrees as the stimulation frequency increases. The 3 dB cut-off frequency was 0.4 kHz (apex) and 7.3 kHz (base). Note that the cut-off frequency of the basal OHC is more than an order of magnitude higher than was previously believed. When the full transducer current was used, the maximum receptor potential was 50 mV (apex) and 35 mV (base). When stimulated at its BF (broken lines, Fig. 3), the OHC receptor potential lagged the transduction current by 64 (apex) and 69 (base) degrees. Our model parameters result in different filtering frequencies for the OHC hair bundle and for the cell body. Thirdly, the hair bundle was mechanically stimulated and the receptor potential was measured in order to observe the combined effect of hair bundle transduction and membrane current (Fig. 3C, D, solid lines without marker). The hair bundle was oscillated with amplitudes of 3.3 and 1 nm (at apex and base respectively) at different frequencies and the receptor potential was observed. The hair bundle displacements were chosen to yield ∼5 percent amplitude change of transducer current that is comparable to the first two simulations. The receptor potential lagged the hair bundle displacement by 49 degrees (apex) and 96 degrees (base). Because the kinetic step between the membrane voltage change and ensuing somatic motility is very fast (>50 kHz, [15]), these phase relations can be considered to be the relations between the hair bundle mechanical stimulation and the OHC somatic reaction. To summarize, a single OHC responded like a low pass filter with a half-power frequency that was lower than the BF of the location. The sharpness of tuning contributed by the transduction apparatus is meager but, nevertheless, this affects the phase difference between the hair bundle displacement and the receptor potential of the OHC.

### Response of the Cochlear Partition to Pure Tone Stimuli

**Figure 4:**
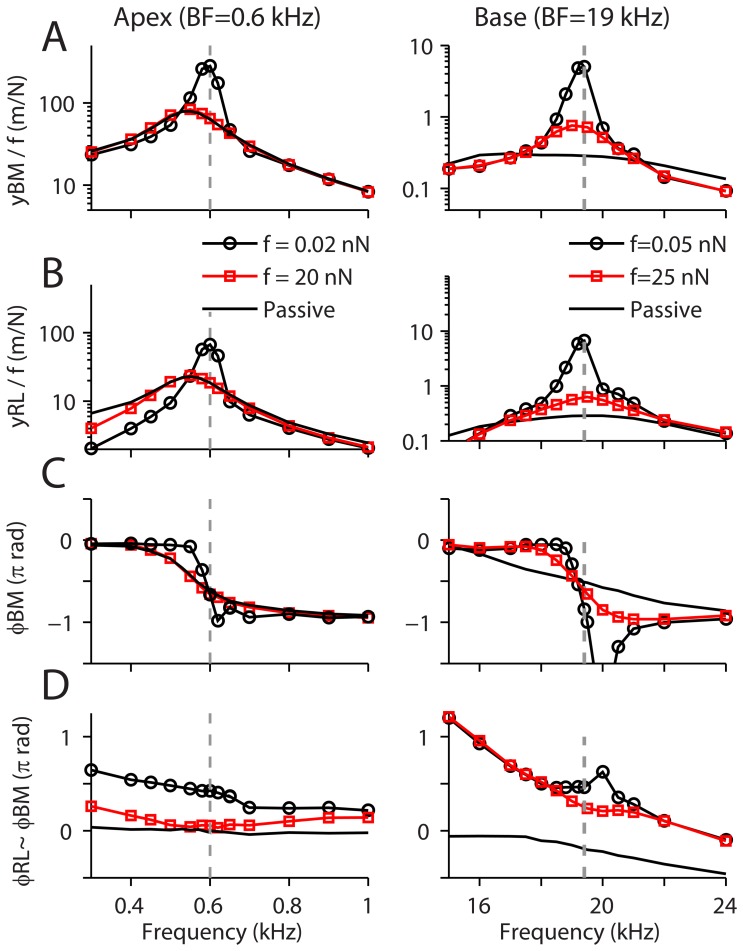
Response of cochlear partition to pure tone stimuli. A sinusoidal force was applied to the basilar membrane (centered at *z* = 0 with normal longitudinal distribution *σ_z_* = 100 µm). Three cases were simulated: stimulation with small and large force (lines with circle or square symbols) and without active feedback from OHCs (solid lines). (**A**) Basilar membrane displacement normalized by applied force. (**B**) Reticular lamina displacement in the y-direction normalized by the applied force. (**C**) Phase of the basilar membrane displacement with respect to the applied force. (**D**) Phase of the reticular laminar y-displacement with respect to the basilar membrane displacement. Note the responses to large stimuli approximate the passive condition in which active OHC feedback is absent.

The apical and basal cochlear partitions were stimulated with sinusoidal force applied to the basilar membrane (Fig. 4). With a small stimulating force, the partition movement peaked at frequencies of 0.60 kHz (apex) and 19.5 kHz (base). The amplification is close to 30 dB at the base and 10 dB at the apex. As the stimulus level was increased, the importance of amplification declined and the responses approached the passive condition. At the apex, the BF shifted to 0.55 kHz as the simulating level increased. At the base, the basilar membrane response of the passive OC had two peaks, one at ∼17 kHz and the other at ∼20 kHz, which correspond to the resonant frequencies of the impulse response (Fig. 2C,D), with the lower frequency peak being dominant. The response of the reticular lamina for the passive basal cochlear partition peaked at ∼ 20 kHz. As a result, in the intermediate stimulus level, the basilar membrane response peaked at a slightly lower frequency than the reticular lamina, agreeing with recent observations [7].

The basilar membrane vibrated in phase with the stimulus at low frequencies below the BF and lagged the stimulus by 180 degrees at frequencies above BF (Fig. 4C). In the passive case, the reticular lamina was in phase with (apex) or lagged (base) the basilar membrane. When the OHC mechanical feedback was turned on, the reticular lamina displacement led that of the basilar membrane (Fig. 4D). The extent of the phase lead decreased as the stimulus frequency and level increased. At the apical cochlear partition, the phase difference (at 0.6 kHz) decreased from 76 to 10 degrees as the stimulus level increased 1000 -fold. At the base, the reticular lamina-basilar membrane phase difference (at 19.5 kHz) decreased from 83 to 43 degrees as the stimulus level increased by 500 times. These results indicate the phase relations between the reticular lamina and basilar membrane during basilar membrane vibration depend crucially on the action of the somatic motor. For low stimulus levels, when the contribution of the OHC somatic motility is significant, the reticular laminar leads the basilar membrane motion by about 90 degrees.

### Inappropriate OHC Membrane Properties Disable the Amplification by the OHCs

**Figure 5:**
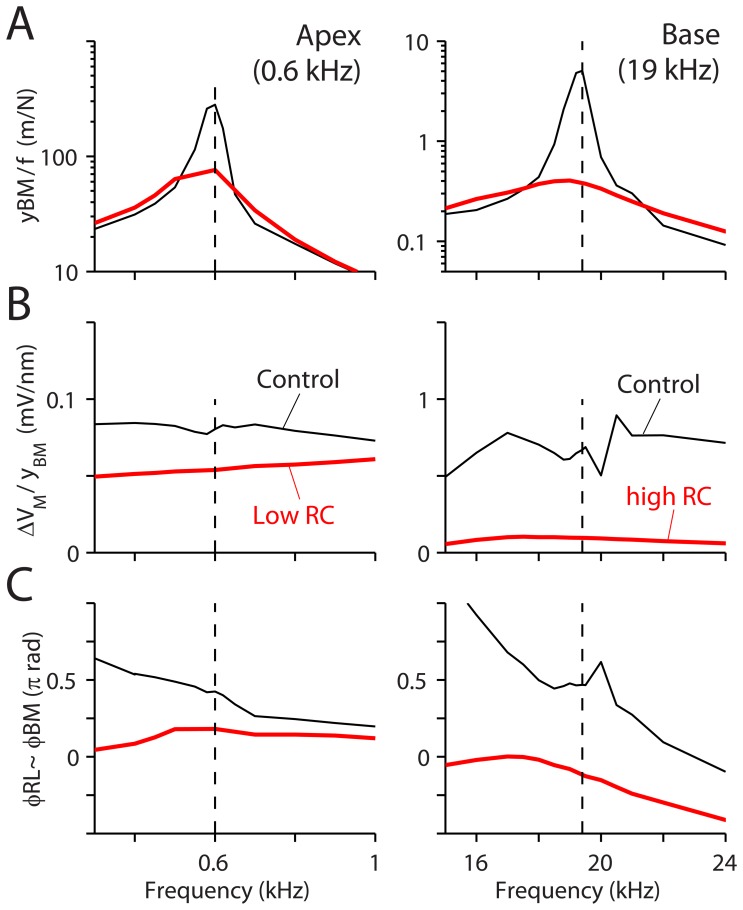
Effect of inappropriate electrical properties of the outer hair cell membrane. The basolateral membrane electrical properties (conductance and capacitance) of the OHC at the apex and the base were exchanged while all other conditions remained the same. The cochlear partitions from the apex and the base (left and right column respectively) were stimulated with pure tones. (**A**) Basilar membrane displacement normalized by applied force. (**B**) Receptor potential of an OHC in the middle of the simulated section normalized by BM displacement. (**C**) Phase difference between the RL and the BM displacement. Thick curves are from tested (with exchanged membrane properties) and the thin curves are from control cases.

To test the importance of the OHC membrane electrical properties for amplification, these membrane properties were exchanged between the apical and basal cochlear partition models (Fig. 5). In other words, the OHC membrane capacitance and conductance measured at the base were assigned to the apex and vice versa. As a result, the OHC membrane RC filtering frequency was higher (apex, 7.3 kHz) or lower (base, 0.4 kHz) than the resonant frequencies of the cochlear partitions. In both cases, the responses became close to those of the passive system. However, the reason for disabling the amplification is different at the two locations. When the membrane electrical time constant was too high (apical OHC with basal properties), despite a comparable receptor potential to the control case (Fig. 5B-apex), the incorrect phase difference between the RL and the BM motion (32 degrees, Fig. 5C-apex) prevented amplification. The incorrect phase relation is ascribed to a reduced phase lag between the OHC transduction current and the receptor potential (from 64 degrees to nearly zero degrees). When the membrane electrical time constant was too low (basal OHC with apical properties), the OHC receptor potential was greatly reduced due to low pass filtering (Fig. 5B-base). As a result, too small OHC a somatic force was recruited to amplify the vibration. This result implies that there exists an optimal range of OHC membrane electrical characteristics and the OHC membrane conductance should increase with the BF in order for the OHCs to fulfill their role as an amplifier, which agrees with the experimental data [10].

**Figure 6:**
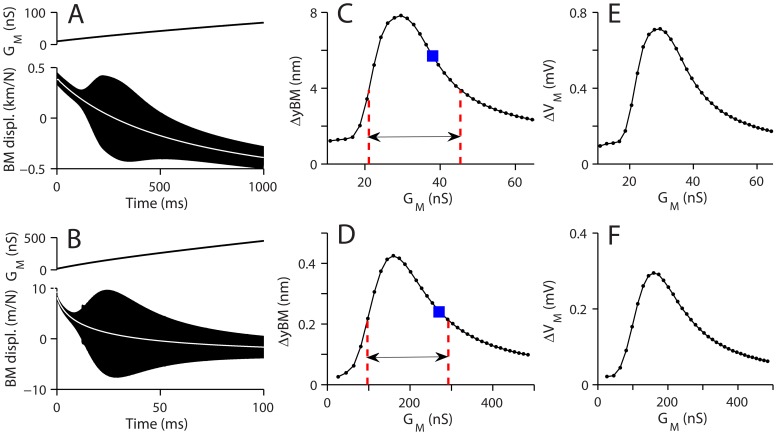
Optimal OHC membrane conductance to amplify basilar membrane vibrations. The basolateral membrane conductance of the OHCs was increased along time axis, while pure tone stimulation was applied. (**A**) The apical section was stimulated with 0.6 kHz 20 pN amplitude force applied at the basilar membrane. During 1000 ms simulation period, the OHC membrane conductance was increased linearly with time from 10 nS to 70 nS (upper plot) and the basilar membrane displacement normalized with the force amplitude was plotted (bottom plot). The stationary position of the basilar membrane (white line) decreased as the membrane conductance increased. (**B**) The basal section was stimulated with 19.5 kHz 50 pN force. During 110 ms simulation period (100 ms shown), the OHC membrane conductance was increased from 30 nS to 480 nS (upper plot). The normalized basilar membrane vibration was plotted (bottom plot). (**C**) Basilar membrane vibration amplitude versus OHC membrane conductance (*G_M_*), apex. Two vertical broken lines indicate 3 dB bandwidth of the *G_M_*. Filled squares (▪) indicate the response with the *G_M_* value used in this study. (**D**) Basilar membrane vibration amplitude versus *G_M_*, base. (**E**) Receptor potential versus *G_M_*. (**F**) Receptor potential versus *G_M_*, base.

The importance of the electrical properties was further explored by asking whether there exists an optimal range for the OHC basolateral membrane conductance (*G_M_*) which is conferred by voltage-dependent K^+^ channels. The apical and basal cochlear partitions were stimulated with sinusoidal force applied to the basilar membrane (0.6 and 19.5 kHz respectively), and siumulaneously the conductance of the OHC basolateral membrane was increased with time (Fig. 6). For the apical model, the maximum conductance *G_M,max_* was increased from 10 to 80 nS during the 1000 ms simulation and for the basal model, it was increased from 18 to 800 nS in 106 ms. The membrane potential changed from −7 to −50 mV at the apex and from −3 to −60 mV at the base. Due to its voltage dependence, *G_M_* increased from 11 to 67 nS at the apex and from 30 to 480 nS at the base (Fig. 6A and B). As the conductance was increased, there was a steady deflection of the basilar membrane towards the scala tympani and the vibration amplitude varied non-monotonically (Fig. 6A and B). The basilar membrane vibration was greatest when the *G_M_* was 30 nS and 160 nS for the apex and the base respectively (Fig. 6C and D). The 3 dB band of the *G_M_* was 21–45 nS for the apex and 97–292 nS for the base (double arrow). Our default *G_M_* value is within this range (indicated with ▪). Variation in the OHC receptor potential with change in *G_M_* showed a similar trend to the basilar membrane displacement (Fig. 6E and F). This result indicates that there is an optimal range of OHC basolateral membrane conductances to achieve cochlear amplification, and that the optimal conductance value is higher at the high BF location which agrees with experimental observation [10].

Because the mechanical and electrical data on the gerbil cochlea are available only up to about 19 kHz, it was not possible to perform experiment-based simulations at higher frequencies. However, if existing parameters were extrapolated to the upper frequency limit of the gerbil cochlea, then significant (20 dB) amplification, reliant on the OHC somatic motor, could still be achieved provided the basolateral conductance *G_M_* was also increased. Sharp tuning at a BF of 41 kHz was generated with *G_M_* = 500 nS but was reduced with larger or smaller conductance values. Interestingly, the value of *G_M_* needed for amplification is close to that obtained by extrapolating the electrical measurements [10]. This suggests that the same principle of optimizing the electrical properties will apply even at the most basal locations in the rodent cochlea.

### Kinematic Gain of the OC

**Figure 7:**
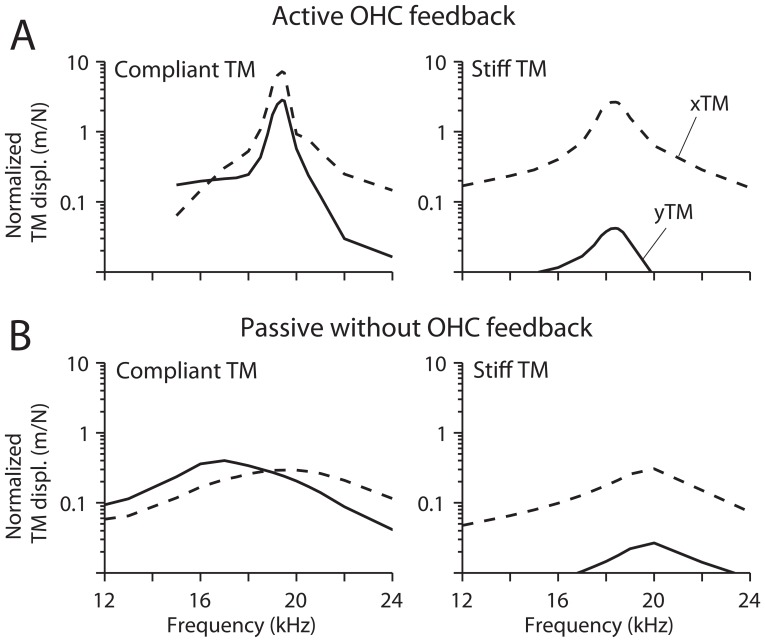
Effect of tectorial membrane stiffness for active and passive responses at the base. (**A**). Active case with OHC feedback. The frequency response of the cochlear partition for a sinusoidal force applied to the basilar membrane for compliant (left) and stiff (right) tectorial membrane. The radial (*x*, solid lines) and transverse (*y*, broken lines) displacement of the tectorial membrane are plotted against stimulation frequency. (**B**) Passive case without OHC feedback. Stimulation conditions and line forms are the same as in (A).

Because of its detailed structural and electrical representation, our fully-deformable finite element model provides more information than other lumped parameter models obtained through kinematic analysis. OC mechanical and electrical responses per 1 nm basilar membrane displacement are summarized in Table 2. Interestingly, active feedback by the OHCs did not improve the kinematic gain of the OC structures such as the displacement of hair bundle, reticular lamina and tectorial membrane per unit basilar membrane displacement. This small kinematic gain is ascribed to a compliant tectorial membrane (comparable to the lower bound of reported values such as [16], [17]). However, we previously found [9] that the kinematic gain of the OC depended on the tectorial membrane stiffness. Fig. 7 shows that the dynamic responses of the OC are different depending on whether a stiff or compliant tectorial membrane is assumed. With the former, the transverse motion predominated, whereas the radial and transverse displacement amplitudes were comparable with a compliant tectorial membrane. In this study, the compliant tectorial membrane was used for two reasons. Firstly, despite a smaller kinematic gain, the OC with a compliant tectorial membrane results in a more sharply tuned motion with active OHC feedback. Secondly, the shift in BF with the action of the OHCs corresponds better to experimental observations when the tectorial membrane is compliant, the OC response peaking at a higher frequency when the OHC somatic motor is functional.

**Table 2:** OC responses per 1 nm basilar membrane displacement.

	Apex (0.6 kHz)		Base (19 kHz)	
	Active	Passive	Active	Passive
*x_HB_* (nm)	0.086	0.097	0.60	0.85
*y_RL_* (nm)	0.24	0.29	1.4	1.0
*x_TM_* (nm)	0.38	0.53	0.59	0.83
*y_TM_* (nm)	0.26	0.32	1.5	1.0
*I_MT_* (pA)	6.8	–	640	–
Δ*V_M_* (mV)	0.080	–	0.72	–
*f_MET_* (pN)	0.068	–	6.5	–
*f_OHC_* (pN)	8.0	–	68	–

*x_HB_* = hair bundle shear displ., *y_RL_* = y-displ. of the reticular lamina, *x_TM_* = x-displ. of the tectorial membrane, *I_MT_* = transduction current of hair bundle, Δ*V_M_* = receptor potential, *f_MET_* = hair bundle force, *f_OHC_* = OHC somatic force.

## Discussion

### OHC is a Phase Maker for the Cochlear Amplification

The OHC somatic motility imposes a phase reversal between the basilar membrane and the reticular lamina [3], [9] sometimes referred to as ‘negative feedback’ [18]. This opposing motion between the top and the bottom surface of the OC attributable to OHC somatic motility combined with the phase lag due to hair bundle motility (Fig. 3) results in ∼90 degree phase difference between the reticular lamina and the basilar membrane. When there is no OHC motility, the reticular lamina motion is in phase with or lags the basilar membrane. As the sound pressure level increases, the contribution of OHC motility compared to the pressure difference across the cochlear partition decreases. Therefore, the opposing motion between the basilar membrane and the reticular lamina becomes less prominent which explains why the reticular lamina phase lead decreases as the stimulus level increases. This phase relation is essential for the OHCs to amplify the vibrations of the cochlear partition. When the polarity of the OHC motility was reversed so that the depolarization of the OHC produced elongation (rather than contraction), the amplification completely disappeared. The OC is often treated as a black box in theoretical studies and the phase relation between cochlear partition displacement (usually represented by the basilar membrane displacement) and OHC active force is assumed. Several models require a tightly restricted phase (timing) for the OHC feedback force to achieve the amplification [18], [19], [20], [21] because only certain phase relations were found effective to counter the viscous energy dissipation in the cochlear partition.

Our results indicate that, in order to achieve amplification, there is an optimal range of OHC membrane time constants according to location (Fig. 5). If the membrane filtering frequency is higher than the BF at the OHC location, the OHC cannot exert its force with correct timing (90 degrees ahead of the basilar membrane movement, Fig. 3). On the other hand, if the membrane filtering frequency is too low, despite the correct phase, the receptor potential becomes too small to generate sufficient force for cochlear amplification. Recently measured membrane properties [10] place the OHC somatic motility within the optimal zone along the cochlear coil. We tested this by applying basal membrane properties to apical cochlear partition simulation and vice versa. For both cases, the amplification entirely disappeared.

### OHC Electrical Properties and the Cochlear Amplification

There has been a concern that the RC filtering frequency of OHC basolateral membrane (<1 kHz) may make high frequency sound amplification difficult [22]. Despite significant force reduction due to the membrane filtering, however, theoretical studies indicated that the OHC somatic force can amplify high frequency sounds with different mechanisms: by trading the gain with the frequency bandwidth [18], by exploiting the extracellular voltage difference between the OC fluid space and the scala media [23], by compensating the membrane filter with longitudinal K^+^ current [24] or by incorporating a large mechano-transduction current that generates large enough receptor potential [25]. These studies assumed the OHC basolateral membrane filter frequency of about 300 Hz, which is more than one order of magnitude lower than the filter frequency at high frequency location in this study (7.3 kHz at 19 kHz BF location, Fig. 3). It is unclear how these cochlear amplification theories would work if they were to incorporate a high filter frequency for the OHC membrane. Perhaps, such a high filter frequency for the OHC membrane may result in even stronger amplification. Otherwise, with too strong feedback from the OHCs, the OC system can become unstable (oscillating in the absence of stimulation). A recent study examined the higher filter frequency of OHC basolateral membrane [26] and found no significant difference from the responses with the slow membrane properties. Another theoretical paper has addressed the significance of the OHC membrane time constant for cochlear function and concluded that the OHC membrane filter frequency and the BF do not have to perfectly match to achieve amplification [27].

Unlike the existing studies [18], [23], [24], [25], our model with the low cutoff frequency of OHC basolateral membrane does not amplify the vibrations of the cochlear partition (Figs. 5 and 6). This difference between our model and others can be ascribed to several reasons. Firstly, our model does not simulate macro mechanics of the entire cochlear coil (e. g., the traveling wave). However, the inclusion of macro mechanics may not change our conclusion as long as the amplification occurs within a finite section of the cochlear coil [28]. Secondly, our present model does not include the effect of the extracellular OC potential, though the experimentally measured OC potential of ∼0.1 mV per 1 nm basilar membrane displacement [29] is small compared to our simulated OHC receptor potential of 0.7 mV per 1 nm basilar membrane displacement (Fig. 5). Furthermore, other studies found that the contribution of the OC potential is minor [24], [25]. Thirdly, we modeled the OC with a fully deformable system, which is different from lumped parameter models of the OC. It is possible that existing lumped parameter models facilitate amplification as compared to a fully deformable OC.

### Critical Value of Mechano-transduction Sensitivity: Single Channel Gating Force

The single-channel gating force (defined as the product of gating swing and stiffness of the transducer channel) determines the sensitivity of the mechano-transduction, *i.e.*, greater single channel gating force results in more sensitive transduction. The single channel gating force used in this study is 4.8 pN which is close to an experimentally estimated value (6.0 pN in [30]). The single channel gating force sets the sensitivity of both the hair bundle force and the somatic force. Our chosen value of 4.8 pN is the value that makes the modeled cochlear partition marginally stable. A larger single channel gating force resulted in instability. This critical value of single channel gating force is also dependent on the magnitude of assumed damping. We chose the damping level so that the passive system is slightly under-damped (Fig. 2D). When the OC and the tectorial membrane were assumed to be rigid bodies, the viscous friction between the tectorial membrane and the reticular lamina could be considered the major energy dissipation mechanism (e.g., [31]). However, it is now believed that both the OC and the tectorial membrane are deformed comparable to the shear motion between the two surfaces. Therefore, correct estimation of the energy dissipation due to the deformation of the OC and the tectorial membrane is required in order to derive the critical single channel gating force. Recent measurements of the dynamic response of the tectorial membrane [32] and the OC [7], [8], if combined with proper mechanical analysis, could be used to estimate the energy dissipation in these structures.

### Stiff Versus Compliant Tectorial Membrane

In our previous work [9], we reported that in order for the OHC somatic and hair bundle force to displace the basilar membrane effectively, a stiff tectorial membrane is beneficial. However, the present results indicate that for sharp tuning, a stiff tectorial membrane is not a necessary condition. While the OC with stiff tectorial membrane (200 kPa Young’s Modulus at the base, equivalent to 8 times the OHC hair bundle stiffness) does not hinder amplification by OHCs, the OC with stiff tectorial requires greater single-channel gating force or higher sensitivity of OHC force generation in order to achieve comparable amplification to the OC with compliant tectorial membrane (10 kPa at the base, equivalent to 0.4 times the OHC hair bundle stiffness). The stiff tectorial membrane condition corresponds to the upper limit of reported value [33] whereas the compliant tectorial membrane condition agrees better with other measurements [16], [17].

Besides the efficiency of amplification, there is another notable difference between the OC with stiff and compliant tectorial membrane. The OC with a stiff tectorial membrane is tuned to a lower frequency when there is OHC force feedback than when it is passive (17 kHz versus 19 kHz at the base; 0.6 kHz versus 0.8 kHz at the apex, Fig. 7). In comparison, the OC with compliant membrane is tuned at a higher frequency when it was active (19.5 kHz versus 16 kHz at the base; 0.60 kHz versus 0.55 kHz at the apex). Experimental results have shown that the resonant frequency of the basilar membrane increased at lower sound pressure levels [34], which is consistent with our simulation using a compliant tectorial membrane. However, this level dependent shift of peak frequency could also be a consequence of fluid-structure interaction in the cochlear duct which was not incorporated in the present model.

## Methods

### Finite Element Model of the Cochlear Partition

The gerbil cochlea sections at 0.6 and 19 kHz BF (about 9.6 and 2.4 mm from the basal end) were chosen for the creation of full 3-D finite element models of the OC. Most structurally significant cells in the OC such as pillar cells, OHCs and Deiters cells have long and thin shapes whose primary direction can be clearly defined (see Figure 1). Acellular structures such as the basilar membrane and the tectorial membrane have obvious orthogonality because of unidirectional collagen fibers running radially. Therefore, the OC structures are represented by beam elements, which allow axial and bending deformation (Fig. 1A). The basilar membrane and tectorial membrane are also represented by a meshwork of beam elements arranged in the radial and longitudinal directions. Their orthogonal micro-structure is explicitly represented by assigning different elastic moduli for the radial and longitudinal elements. The hair bundles are represented by a hinged link between the reticular lamina and the tectorial membrane. A rotational spring at the bundle rootlet has the equivalent shear stiffness of the hair bundle. The reticular lamina is also represented by radial and longitudinal beams. Along the longitudinal direction the arrays of radial sections repeat every 10 µm. This stack of radial sections is combined in the longitudinal direction by four different elastic structures. Three of them are continuous–longitudinal beams of basilar membrane, reticular lamina and tectorial membrane, while the coupling by the OHC and Deiters cell process complex is discrete like the truss structure of a bridge. The OHCs and Deiters cells are tilted in the opposite directions–toward base and apex respectively. Considering the longitudinal space constants of the basilar membrane at the apex and the base [14], longitudinal sections with span of 600 and 900 µm at the base and apex were considered enough to insure that the response in the middle of simulated patch is free from discontinuous boundary conditions. Geometrical properties were obtained from known anatomical data (Table S2 in Supporting Information S1).

The mass of the finite element model were determined as follows. The mass density of all the structural components, including the basilar and tectorial membranes, the hair cells, pillar cells and Deiters cell, were assumed to be that of water (1.0 kg/L). In the finite element model, components that might bear little mechanical load were omitted. Firstly, the thickness of the basilar membrane in Table S2 in Supporting Information S1 represents the collagen fiber layer thickness which is thinner than true basilar membrane including the ground substance. Secondly, non-structural supporting cells in the OC (e.g., Hensen’s cells and Claudius cells) were not included in the finite element model. Finally, the fluid mass carried by the tissue was not explicitly included. These result in underestimate of the mass. To compensate for that, mass components were incorporated into the model as the ‘mass’ thickness of basilar membrane. The mass thickness of the basilar membrane, which was 100 µm at the apex and 40 µm at the base, was chosen for the model to approximately match the BF of that section.

Viscous forces were assumed to consist of two components: the damping within the cleft between the tectorial membrane and the reticular lamina, and the viscous resistance acting on other parts of the organ of Corti. The first component was calculated as the viscous force between two parallel plates of separation *d* immersed in a Newtonian fluid of viscosity 0.72 mN m^−2^s. This yields frictional coefficients per unit cochlear length (along the z-direction) of 0.01 and 0.03 N s m^−2^ at apex and base respectively. This friction between two layers was lumped with that of the hair bundles, acting against their shear direction. The damping of other structures was obtained by multiplying the stiffness matrix by a constant to match the simulated step responses to experimental results, which was equivalent to structural damping per unit length of 0.03 and 0.07 N s m^−2^ for apex and base respectively.

### Mechano-transduction Channel Kinetics

Mechano- transduction in the OHC bundle is modulated by the relative displacement between the tectorial membrane and the reticular lamina which equals the hair bundle shear displacement (x_HB_). The transducer channel kinetics are based on our previous work [30], [35]. See Table S1 in Supporting Information S1 for parameter values. There are ten states–two states depending on closed or open configuration multiplied by five states depending on the number of calcium bound. Fast adaption is determined by calcium binding to the channel. The probability of calcium binding to the channel *p_B_* is described by
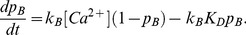
(1)Where, *k_B_* is a rate coefficient, [*Ca^2+^*] is the calcium concentration at the transduction channel and K_D_ is the dissociation coefficient. The channel open probability *p_o_* is defined by
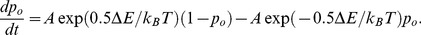
(2)Where, the energy difference between the two channel states, ΔE, is a function of hair bundle shear displacement xHB and the number of calcium bound nCa.
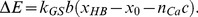
(3)Where, kGS is the stiffness of putative gating spring, x0 is a constant set by myosin motors and c is the channel’s morphological change due to calcium bind. A positive value of c implies that calcium binding to the channel facilitates the channel closure and stabilizes the closed state [36], [37]. Then the force exerted by a hair bundle to its external system, fMET, is
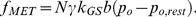
(4)


### OHC Electrical Circuit

The K^+^ ions in the endolymph space (scala media) enter into the IHCs and OHCs as the mechano-transduction channels in the hair-bundles are opened. The driving battery of such an ionic flow is the sum of the cells’ resting membrane potential and the endocochlear potential. While the endocochlear potential is strongly maintained by the stria vascularis, the OHC membrane potential fluctuates according to the activation of the mechano-transduction current. The resting membrane potential is predominantly determined by the equilibrium between the mechano-transduction current (inward) through the hair-bundle and the K^+^ current at the basolateral membrane (outward). For OHC electrical properties, the values from gerbil and rat cochlear are used [10]. Nonlinear OHC somatic motility is represented by coupling the OHC electrical circuitry with the OHC mechanics (Fig. 1B). The electrical representation of the OHC has two parts: the apical part represents the hair-bundle’s mechano-transduction (conductance *G_S_* and current *I_MT_*); the basal part represents the lateral membrane (collective K^+^ conductance *G_M_*, K^+^ equilibrium potential *E_K_*, linear capacitance *C_M_* and current by piezoelectric charge movement *I_C_*), which can be expressed as
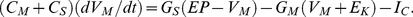
(5)The membrane conductance *G_M_* is voltage and time dependent.
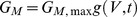
(6)Where,



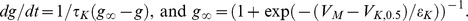
The conductance of the stereocilia GS is proportional to the transduction channel open probability *p_o_*.



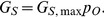
(7)Following the existing two-state membrane theory [21], [38], [39], [40], the charge incorporated with the piezoelectric current (*I_C_ = dQ/dt*) is
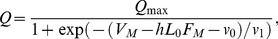
(8)Where, Q_max_ is the maximum nonlinear charge moving across the membrane, L_0_ is the original (unconstrained) length of the OHC, F_M_ is the membrane tension and h is the piezoelectric coefficient defined as
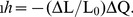
(9)ΔL is length change of the OHC when unconstrained. The membrane tension is obtained from
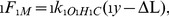
(10)where kOHC is the axial stiffness of the OHC and y is the elongation of the OHC. They is obtained from the OC mechanics such as
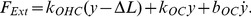
(11)
Equations (7)–(9) are used when we simulate a single OHC response (Fig. 1B). When simulating in the full scale finite element model, the OHC membrane force is generated by changing the original length using Eq. (9). The complete set of model parameters is presented in the supplementary material.

### Computation

The program was written in Matlab (ver. 7.11, MathWorks). No Matlab toolbox was used. The code was run on an IBM PC (Intel Xeon processor, 2.4 GHz, 12 GB RAM). Typical time step size for the integration of differential equations was 10 and 1 µs for the apical and the basal section respectively. The fastest kinetics was the transducer channel activation that prevented us from using greater time step size. It took about 2 minutes to simulate 1 ms response of the basal partition model. The computer code is available upon request.

## Supporting Information

Supporting Information S1 Supporting tables.
(PDF)
