# Supplementary material for: Optimal Electrical Properties of Outer Hair Cells Ensure Cochlear Amplification
Source: PLoS One. 2012 Nov 27;7(11):e50572. doi: 10.1371/journal.pone.0050572 (PMC3507780; doi:10.1371/journal.pone.0050572)
Supplement: Supporting Information S1 — Supporting tables. (PDF) [file pone.0050572.s001.pdf]

# SUPPORTING MATERIALS

**Table S1. OHC Hair bundle mechano-transduction parameters**

| Symbol | | Apex | Base | Description | Reference |
|---|---|---|---|---|---|
| $H$ | (µm) | 5 | 2 | Hair bundle height | 1 |
| $\gamma$ | - | 0.11 | 0.3 | Elongation of the gating spring per unit displacement of hair bundle tip | 2 |
| $A$ | ($ms^{-1}$) | 100* | 100 | Channel activation rate constant | 3 |
| $k_{GS}$ | (mN/m) | 6 | 6 | Gating spring stiffness | 2 |
| $b$ | (nm) | 0.8 | 0.8 | Gating swing | 2 |
| $c$ | (nm) | 0.7 | 0.7 | Ca binding modification | 2, 4 |
| $k_B$ | ($ms^{-1}\mu M^{-1}$) | 0.4 | 0.4 | Ca binding coefficient | 2, 4 |
| $K_D^C$ | (µM) | 20 | 100 | Ca dissoc. const. when a channel is closed | 2 |
| $K_D^O$ | (µM) | 1 | 1 | Ca dissoc. const. when a channel is open | 2 |
| $C_0$ | (µM) | 1 | 1 | [$Ca^{2+}$] near the channel when a channel remains closed | 5, 6 |
| $C_1$ | (µM) | 20 | 100 | [$Ca^{2+}$] near the channel when a channel remains open | 5, 6 |

* For Fig. 6, $A$ = 10 $ms^{-1}$ was used for the apical model.

1. Lim, 1986, Hear. Res.
2. Beurg, Nam et al., 2008, Biophys. J.
3. Ricci., personal communication
4. Nam and Fettiplace, 2008, Biophys. J.
5. Beurg, Nam et al., 2010, J. Physiol.
6. Beurg, Ricci et al., 2009, Nat. Neurosci.

**Table S2**. **Organ of Corti structural properties**

| Structure | Parameter | Apex** | Base** | Reference |
|---|---|---|---|---|
| Basilar membrane* | Width<br>Thickness<br>$YM_X$, $YM_Z$ | 280<br>0.7<br>1000, 0.1 | 160<br>3.8<br>1000, 0.2 | 1, 2, 3 |
| OHC soma | Diameter<br>Length<br>YM | 8<br>50<br>0.015 | 8<br>20<br>0.015 | 4<br>4<br>5, 15 |
| OHC hair bundle | Height<br>Stiffness | 5<br>5 | 2<br>50 | 6, 7, 8, 9 |
| Pillar cells | Diameter<br>YM | 4<br>2 | 8<br>20 | 10, 11<br>12† |
| Deiters cell<br>(base, process) | Diameter<br>YM | 10, 1<br>0.1, 0.5 | 10, 1.5<br>1, 0.5 | 10, 11<br>12† |
| Reticular lamina<br>(tunnel of Corti, OHC) | Thickness<br>$YM_X$, $YM_Z$ | 5, 2<br>0.4, 0.04 | 5, 2<br>4, 0.4 | |
| Tectorial membrane<br>(root, body) | Width<br>Thickness<br>$YM_X$<br>$YM_Z$ | 105, 105<br>25, 50<br>4e-3, 1e-3<br>50e-6 | 40, 40<br>25, 30<br>0.04,0.01<br>500e-6 | 13, 14‡ |

Dimensions are in μm for length, MPa for YM (Young's modulus) and mN/m for stiffness.
*We did not consider the difference between the acuate and pectinate zone.
** All the parameter values have a longitudinal gradient. Even within each apical or basal finite element model, the values are exponentially graded. The values at two points presented here set the gradient.
† The pillar cell has orthogonal property: stiff in the axial direction because of its bundled micro-tubules. Because isotropic property was used for simplicity, the pillar cell in this work has comparable bending stiffness to the orthotropic one, but more compliant in the axial direction.
‡ Values of these YM are from ref#13. The ratio between the body and the root of TM is taken from ref#14.


1. Plassmann, Peetz and Schmidt, 1987, Brain Behav. Evol.
2. Schweitzer, Lutz, et al., 1996, Hear. Res.
3. Edge, Evans et al., 1998, Hear. Res.
4. He, Evans and Dallos, 1994, Hear. Res.
5. Iwasa and Adachi, 1997, Biophys. J.
6. Strelioff, Flock and Minser, 1985, Hear. Res.
7. Lim, 1986, Hear. Res.
8. Roth and Bruns, 1992, Anat. Embryol. (Berl)
9. Beurg, Nam et al., 2008, Biophys. J.
10. Slepecky, 1996, The Cochlea (Ed. by Dallos, Popper and Fay)
11. Angelborg, Engstrom, 1972, Acta Otolaryngol. Suppl.
12. Tolemeo, Holley, 1997, Biophys. J.
13. Gavara, Chadwick, 2009, PLoS One
14. Gueta et al., 2006, PNAS
15. He and Dallos, 1999, PNAS
